# The Impact of Isothermal Treatment on the Microstructural Evolution and the Precipitation Behavior in High Strength Linepipe Steel

**DOI:** 10.3390/ma13030634

**Published:** 2020-01-31

**Authors:** Yong Tian, Hongtao Wang, Xiaoning Xu, Zhaodong Wang, R.D.K. Misra, Guodong Wang

**Affiliations:** 1 State Key Laboratory of Rolling and Automation, Northeastern University, Shenyang 110819, China; tianyong@ral.neu.edu.cn (Y.T.); neu-x.xu@foxmail.com (X.X.);; 2 Laboratory for Excellence in Advanced Steel Research, Department of Metallurgical, Materials and Biomedical Engineering, University of Texas at El Paso, El Paso, TX 79968, USA

**Keywords:** linepipe steel, isothermal holding temperature, microstructural evolution, precipitation, hardness

## Abstract

Isothermal treatment affects the microstructural evolution and the precipitation behavior of high-strength low alloy (HSLA) steels. In this regard, thermal simulation of different isothermal treatment temperatures was adopted by using a thermomechanical simulator. The results showed that hardness reached the maximum value at 600 °C holding temperature, which was related to a finer grain structure and granular bainite. The strengthening effect of precipitates was remarkable due to the combination of small particle size and small interparticle spacing. It is presumed that the precipitation started after 600 s at 600 °C. Precipitation strengthening continued to exist, even though coarsening of ferrite grains led to softening phenomena when the specimen was isothermally held at 750 °C, which led to relatively high hardness. The precipitates were fcc (Ti, Nb) (N, C) particles, and belonged to MX-type precipitates. Average size of precipitates increased from 3.14 to 4.83 nm when the specimens were isothermally held between 600 °C and 800 °C. Interparticle spacing of precipitates also increased with increasing isothermal treatment temperatures. These led to a reduction in precipitation strengthening. At the same time the polygonal ferrite content increased and ferrite grain size got larger, such that the hardness decreased continuously.

## 1. Introduction

High-strength low alloy (HSLA) steels present a good combination of high strength and ductility obtained through the addition of microalloying elements, thermo-mechanical controlled processing (TMCP), and processes capable of producing complex microstructures that improve the mechanical properties of steels [1]. HSLA steels containing niobium and/or titanium as the microalloying elements are widely used for construction, line pipe, pressure vessel, engineering, automobile, naval, and defense applications [2,3,4]. Over the past few decades, the need for improved combinations of high strength, toughness and weldability on an industrial scale at affordable prices has driven the development of steel for production lines [5,6,7]. In order to improve transportation efficiency and safety under high pressure conditions, linepipe steel has become thicker and larger in diameter. The reliability and safety of oil and gas pipelines under various conditions of use and different mechanical damage need to be evaluated due to the higher requirements for structural integrity and environmental factors for safety assessment [4,8]. High-grade pipeline steels have been used for the practical application [9,10,11,12]. Desired mechanical properties can be obtained by optimizing alloy design and using TMCP process [13]. TMCP consisting of controlled rolling followed by controlled cooling, is used to maximize the benefits of the microalloying additions present in microalloyed steels [14]. Application of TMCP leads to the decrease of the effective ferrite grain size and the increases of the dispersed precipitate and the dislocation density, which eventually resulted in the improvement of comprehensive properties such as strength, toughness and weldability. It also improves the morphology of MA (martensite-austenite) constituent, which lowered the yield ratio and, thereby, enhanced the capacity for strain hardening [15]. Microalloying elements can effectively control the microstructure of steel and improve its mechanical properties. Reasonable addition of alloying elements provides excellent heat affected zone (HAZ) toughness and guarantees sufficiently low ductile-to-brittle transition temperature [16]. Nb microalloying is the backbone of HSLA steel metallurgy, providing a favorable combination of strength and toughness by pronounced microstructural refinement [17]. Nb microalloying raises the no-recrystallization temperature, leading to a more pancaked austenite and higher Sv (grain boundary surface area per unit volume) values. Thus, dislocation, deformation zone, and twin boundary increase which leads to a refined ferrite/martensitic microstructure that resulted in a good combination of strength and toughness [18].

For a given material, optimal control thermomechanical process is based on a thorough understanding of possible microstructural evolutions such as recrystallization and precipitation during thermal processing [19]. Microalloying elements, such as niobium, titanium, and vanadium, are precipitated in austenite (γ) as carbides, nitrides, or carbonitrides during hot rolling process, and contribute to the mechanical properties of the steel. The reasons for the performance improvement are grain refinement, solid solution hardening, and precipitation hardening [20]. Nb and Ti carbides/carbonitrides are formed in austenite at high temperature, and V carbides/carbonitrides are precipitated at low temperature. These precipitation control and microstructure evolution control can be achieved by thermomechanical processing [20]. Nevertheless, there are few studies that reports microstructural evolution and precipitation behavior in X90 linepipe steels on different isothermal holding. 

It is well known that the production of linepipe steel includes coiling after controlled rolling and run-out table cooling. Coiling is an approximate isothermal process. Different positions of the steel coil have undergone different isothermal treatment. It is not a long isothermal process for the surface and near surface position of the steel coil. When the coiling temperature for a HSLA steel is 550 °C, a large number of <10 nm nano-scale (Nb,Ti)C precipitates are obtained [21]. The precipitation kinetics of (Nb,Ti)C particles in ferrites depends on the isothermal temperature. There is a competition mechanism for austenitic defects between ferritic transformation and precipitate nucleation. Several kinds of complex carbides during isothermal treatment and their strengthening ability has been verified. However, there are still some precipitates precipitated during isothermal process and the strengthening mechanism which are not clear. The precipitation distributed along the austenite/ferrite interfaces or in the ferrite matrix determines the type of ferrite strengthening. Therefore, systematic studies on the effects of isothermal treatment on microstructural evolution and the precipitation behavior in X90 linepipe steel are needed.

In the present work, the thermal simulation using X90 linepipe steel was conducted. The Vickers hardness distribution after different isothermal treatment temperatures was compared. The characteristics of microstructural evolution and precipitation behavior after different isothermal temperatures were analyzed in terms of microstructural constituents of steel. The purpose is to contribute to a better understanding of the formation of bainitic ferrite (BF), granular bainite (GB), polygonal ferrite (PF), and martensite/austenite (MA) islands, as well as the distribution of the precipitates after different isothermal treatments. Strength contribution of these factors could be explained by considering the Vickers hardness distribution and microstructural evolution after different isothermal temperatures. This research has certain guiding significance for the formulation of different types of pipeline steel production process.

## 2. Materials and Methods 

**Figure 1:**
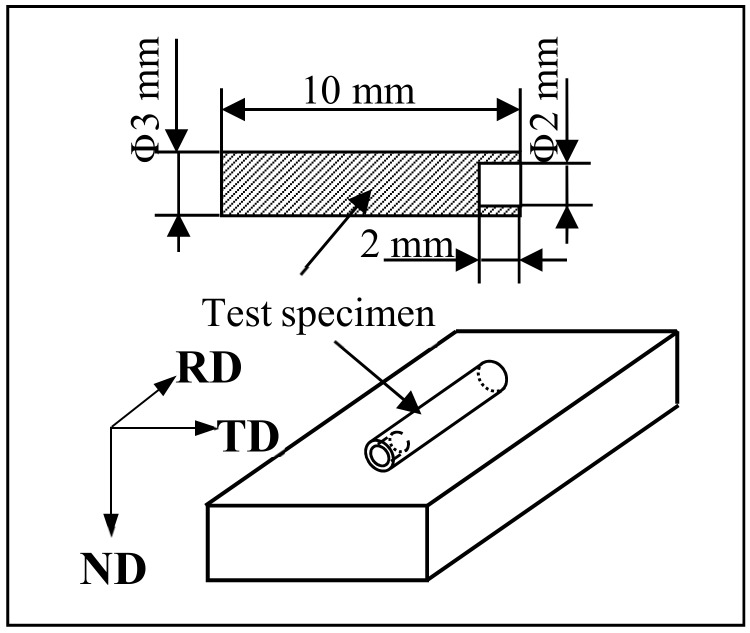
Specimen geometry for thermal expansion testing.

**Figure 2:**
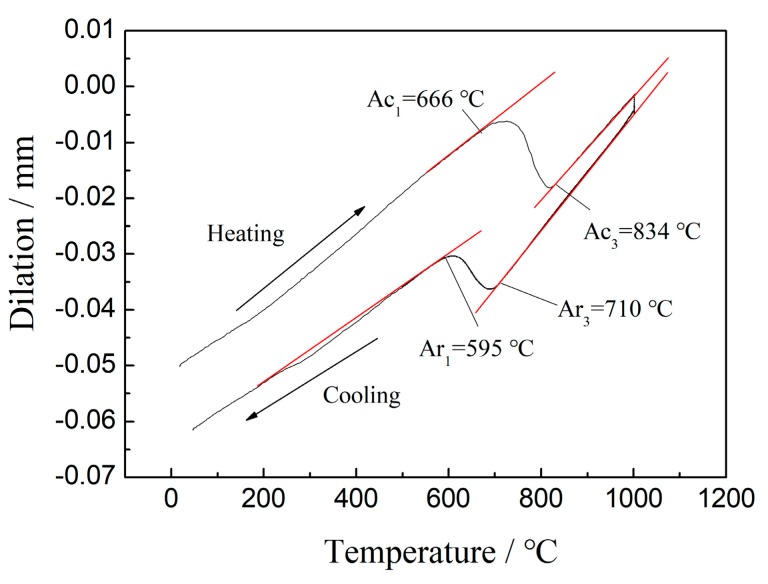
Dilation curves recorded during heating of the experimental steel.

**Figure 3:**
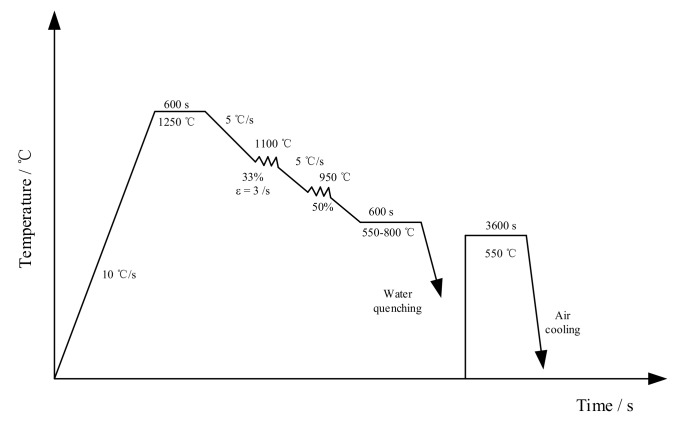
Schematic illustrations describing the thermal simulation.

An API X90-grade steel with a minimum yield strength level of 625 MPa (90 ksi) was used in this study. Its nominal chemical composition is Fe-0.059C-0.0045N-1.870Mn-0.230Si-0.025Al-0.076Nb-0.014Ti-0.005V-0.350Ni-0.240Cr-0.190Cu-0.185Mo wt%. The dilatometry specimens were machined from industrial pipeline steel, and their standard size and shape is shown in Figure 1. The dilatation curve obtained while heating and cooling is shown in Figure 2. The heating and cooling rate for dilatometry test was 30 °C/s and 5 °C/s (The inflexion in the dilatation curve was the transition point). The calculation was performed by the thermodynamic Thermo-Calc software (Thermo-Calc Software AB, Solna, WEDEN) to determine the critical parameters of Ae1 and Ae3. The results were 666 °C and 834 °C, respectively. Ar1 and Ar3 were 595 °C and 710 °C, respectively. Cylindrical specimens with a diameter of 8mm and a length of 15 mm were machined along the transverse direction of the hot-rolled sheet. The cylindrical specimens were subjected to thermomechanical simulation using a Gleeble 1500 thermomechanical test system. Graphical description of the process flow of the thermal simulation test was shown in Figure 3. The specimens were first soaked for 600 s at 1250 °C. The heating process was to simulate reheating of slabs before rolling and to ensure that most of the existing precipitates were dissolved. Then, the heated specimens were deformed to 33% (strain rate 3/s) at 1100 °C and 50% (strain rate 10/s) at 950 °C. Subsequently, the specimens were cooled at the rate of 30 °C/s to 550 °C, 600 °C, 650 °C, 700 °C, 750 °C, and 800 °C, respectively, and isothermally held for 600 s. Finally, the specimens were water-quenched to room temperature for terminating further interphase precipitation (IP). Thereafter, all specimens were heated to 550 °C and held for 3600 s, and the effect of IP on hardness can be detected.

The hardness (HV0.5) of the thermal simulation specimens was measured using a HV-50A hardness tester with a load of 0.5 kgf. The hardness data was measured five times for each process and the standard deviation was calculated. In order to evaluate the hardness data of each phase and MA island, a microhardness tester with a load of 10 gf, and nano-indentation on a Triboindenter with a load of 10 mN were used. Standard abrasive polishing and 4% nitric acid solution etching methods were used to prepare samples for optical microscopy. LePera etchant was used to highlight the microstructural constituent [22]. Optical microscopy observations were conducted with a SSX-550 scanning electron microscopy (SEM) (SHIMADZU Co., LTD, Kyoto, Japan) and an EM 400T transmission electron microscopy (TEM) (Koninklijke Philips N.V., Amsterdam, the Netherlands). TEM specimens were electro-polished with a solution containing 10% perchloric acid in methanol at a temperature of −30 °C and a voltage of 40 V DC. Measurement of volume fraction of microstructural constituents and precipitates was conducted using Leica DMIRM (Leica Microsystems Inc., Buffalo Grove, IL, USA) and the IPP image analyzer (Media Cybernetics, Inc., Rockville, MD, USA).

## 3. Results

### 3.1. Vickers Hardness

**Figure 4:**
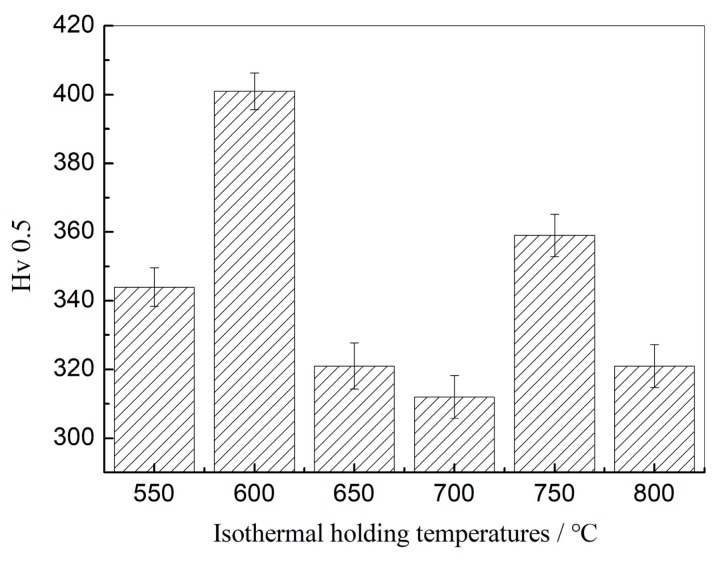
Effect of isothermal temperature on Vickers hardness.

Figure 4 shows the effect of isothermal holding temperature on Vickers hardness of specimens. With the increase of isothermal temperature, the hardness was increased and reached maximum value (HV0.5 401) at 600 °C holding temperature. Above this temperature, the hardness decreased with increased holding temperature, and it reached minimum value (HV0.5 312) at 800 °C holding temperature. The hardness decreased gently with increased holding temperature from 650 to 800 °C.

### 3.2. Optical Microscopy

**Figure 5:**
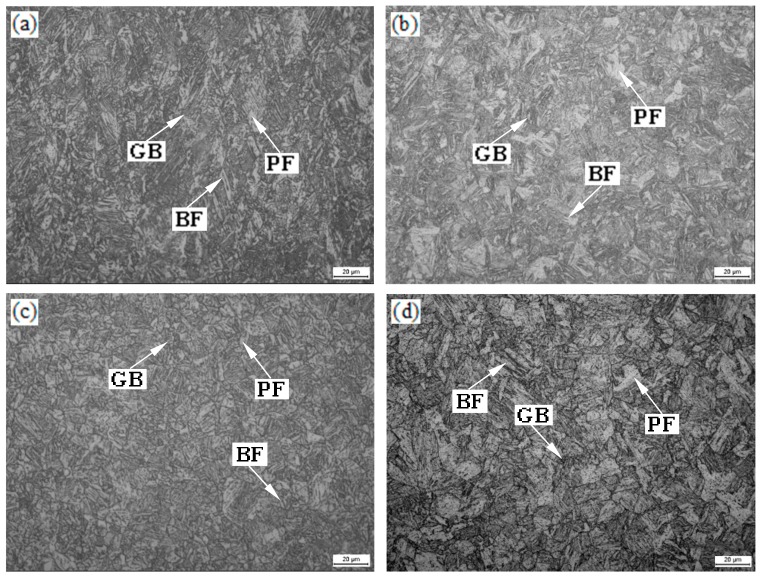

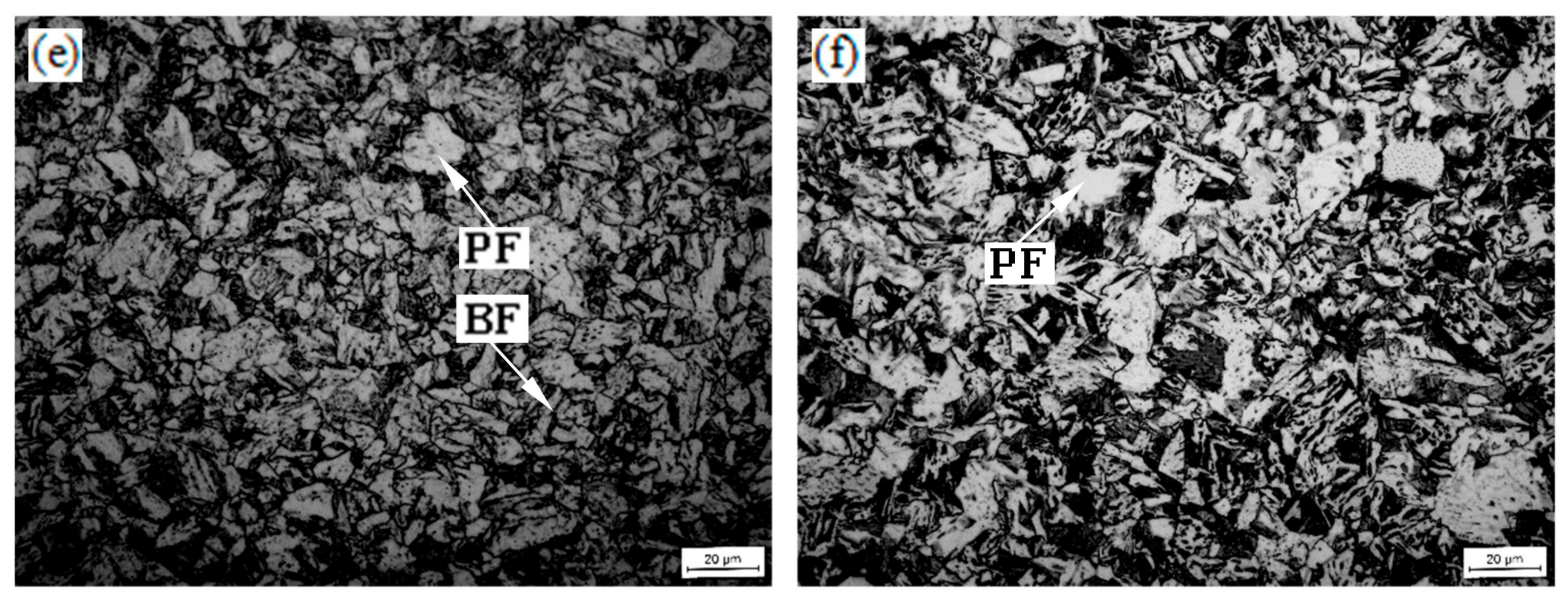
Optical micrographs of the experimental steel (by Nital etched) held at (**a**) 550 °C; (**b**) 600 °C; (**c**) 650 °C; (**d**) 700 °C; (**e**) 750 °C; (**f**) 800 °C.

Microstructure changed at different isothermal temperatures are shown in Figure 5. The microstructure consisted of BF, GB, and PF after different isothermal conditions. Martensite was not present due to tempering. GB and BF were found in all thermally simulated microstructure. The content of PF was the least at 550 °C holding temperature. It increased gradually with increased holding temperature (Figure 5a–c). Coarse PF was obtained at 700 °C holding temperature (Figure 5d). The amount of PF reached the highest value and the coarsest PF grains were obtained at 800 °C holding temperature (Figure 5f).

**Figure 6:**
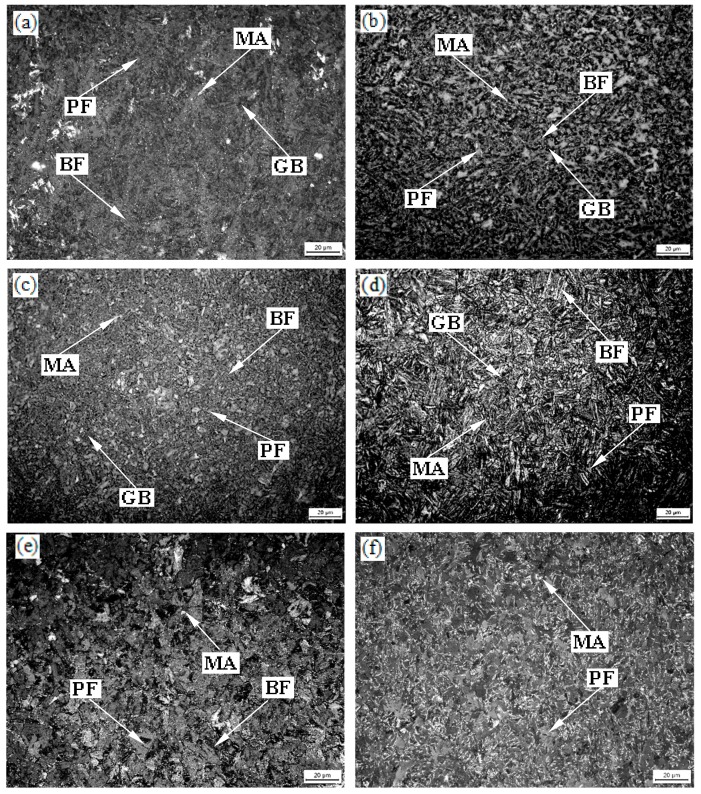
Optical micrographs of the experimental steel (by LePera etched) held at (**a**) 550 °C; (**b**) 600 °C; (**c**) 650 °C; (**d**) 700 °C; (**e**) 750 °C; (**f**) 800 °C.

Optical microstructure for the experimental steel using LePera etchant is shown in Figure 6. The volume fraction of the microstructural constituents and the average grain size of PF are shown in Table 1. After etching, ferrite appeared gray, bainite appeared black, and MA islands appeared white [22]. It could be seen from the figure that PF was less at lower isothermal conditions. The amount of PF increased gradually and coarsened with increased holding temperature up to 800 °C, which is evident in Figure 6f. The amount of MA islands increased at 800 °C holding temperature. The hardness distributions of the microstructural constituents are shown in Table 2. In Table 2, the hardness reached maximum value at 600 °C holding temperature for all phase. The hardness of GB, BF, and PF decreased in proper order as a whole.

### 3.3. Electron Microscopy

**Figure 7:**
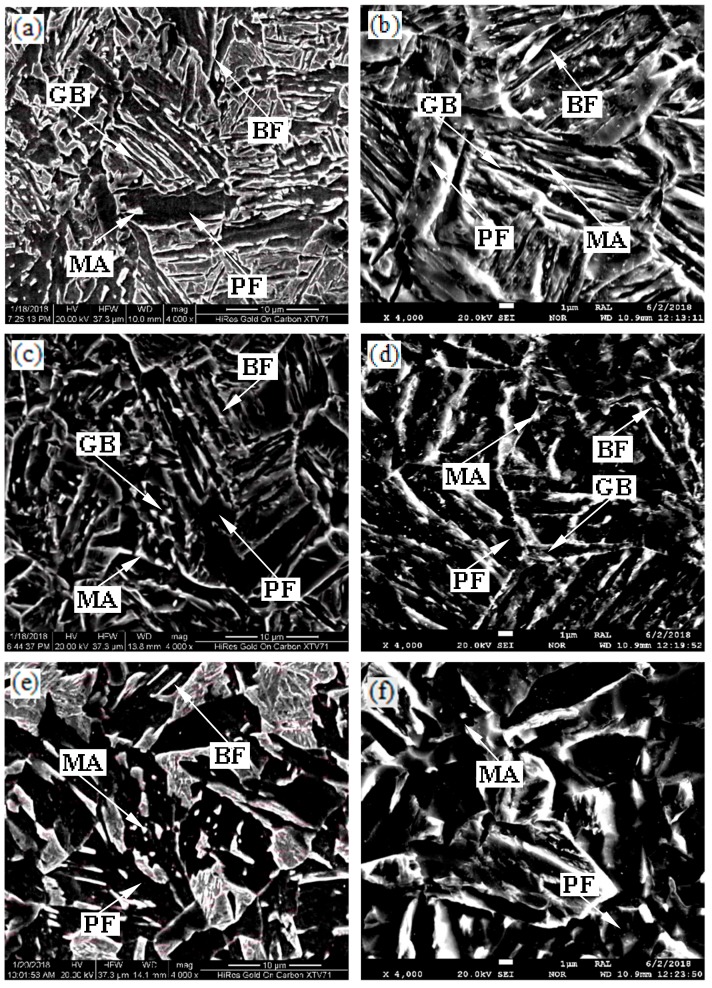
SEM micrographs of the specimens held at (**a**) 550 °C; (**b**) 600 °C; (**c**) 650 °C; (**d**) 700 °C; (**e**) 750 °C; (**f**) 800 °C

Figure 7 shows SEM micrographs of specimens after different isothermal conditions. BF and GB dominated the microstructure at 550 °C holding temperature. GB slightly increased at 600~750 °C holding temperature. White MA islands were obvious at different isothermal holding temperatures. PF grains coarsening was observed with increased holding temperature, which is consistent with optical microscopy observations. 

**Figure 8:**
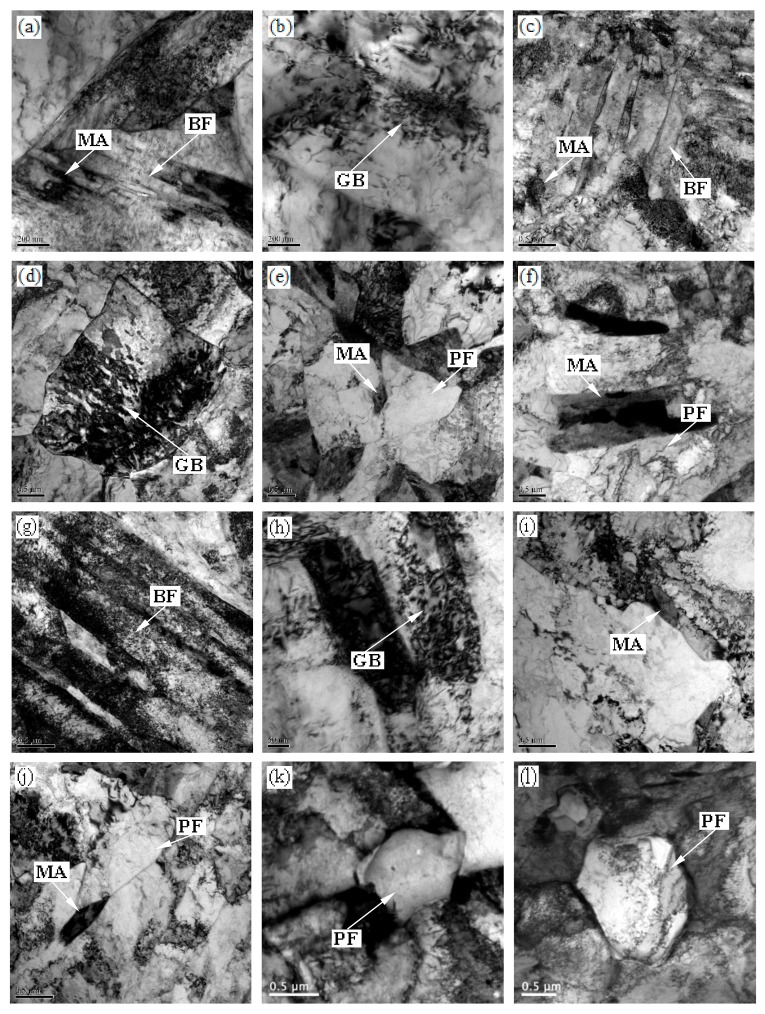
TEM micrographs of the specimens held at (**a**,**b**) 550 °C; (**c**,**d**) 600 °C; (**e**,**f**) 650 °C; (**g**,**h**) 700 °C; (**i**,**j**) 750 °C; (**k**,**l**) 800 °C.

TEM micrographs (bright filed) of specimens after different isothermal conditions are shown in Figure 8. It might be noted that MA islands were present in the middle of BF plate at 550 °C holding temperature (Figure 8a,b). BF plate and MA islands in the bainitic matrix were observed at 600 °C holding temperature (Figure 8c,d)). PF appeared except for MA islands at 650 °C holding temperature (Figure 8e,f). BF plate exhibited lath morphology when the specimen was isothermally held at 700 °C (Figure 8g,h). MA islands were obvious in the bainitic matrix at 750 °C holding temperature (Figure 8i,j). The desired lath morphology was not obvious, and it possessed transformation characteristics of PF at 800 °C holding temperature, as shown in Figure 8k,l.

**Figure 9:**
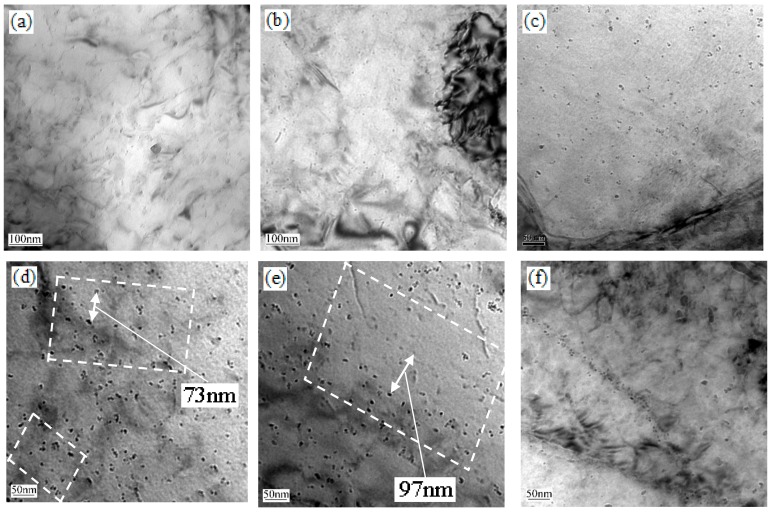
The precipitate particles of the specimens after isothermally held at (**a**) 550 °C; (**b**) 600 °C; (**c**) 650 °C; (**d**) 700 °C; (**e**) 750 °C; (**f**) 800 °C

The morphology in bright filed of precipitates in specimens after different isothermal process is shown in Figure 9. Precipitates were nearly absent, except for a few precipitates that were distributed in the BF matrix when the specimen was isothermally held at 550 °C. The precipitate was spherical or rectangular ~29 × 31 nm. (Figure 9a). Fine dispersed precipitates (Nb/Ti carbonitrides) were observed at 600 °C holding temperature (Figure 9b). Dispersed precipitates increased at 650 °C holding temperature (Figure 9c). These precipitates became coarse at 700 °C and 750 °C holding temperature (Figure 9d,e). Interphase precipitation carbide can be found out in the matrix. Some regular arrays of carbides were bracketed by white broken lines (Figure 9d,e). Relatively coarse carbides were observed in BF matrix (Figure 9f).

**Figure 10:**
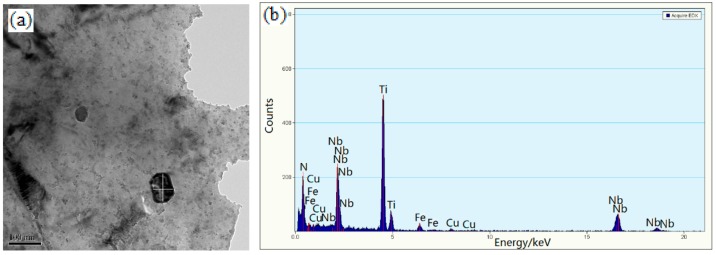

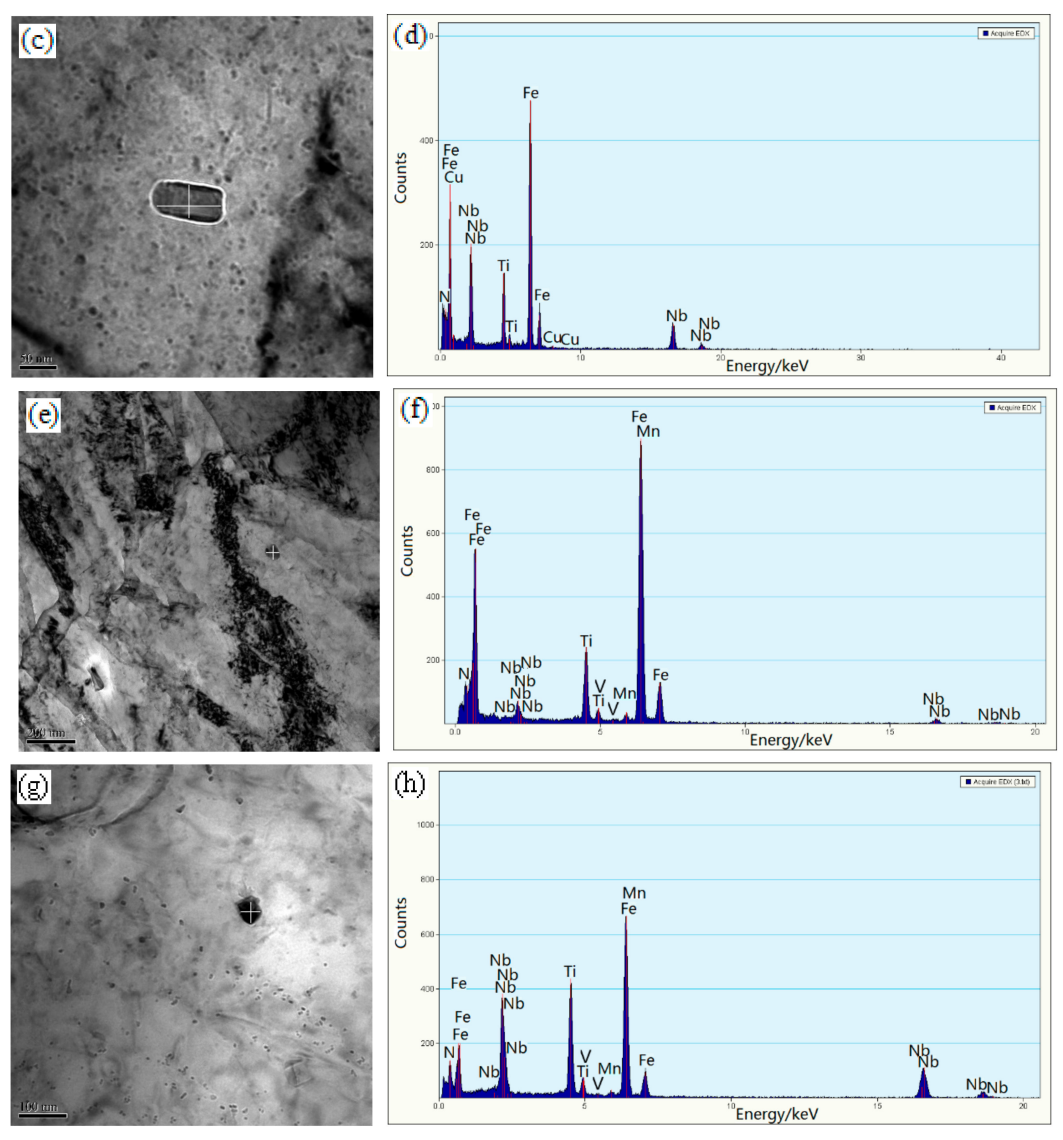
The precipitate particle and its EDS image held at (**a**,**b**) 550 °C; (**c**,**d**) 600 °C; (**e**,**f**) 700 °C; (**g**,**h**) 800 °C

There were a few large-sized precipitates besides fine dispersed precipitates at 550 °C, 600 °C, 700 °C, and 800 °C holding temperatures in Figure 10 in bright filed. They exhibited square-shaped morphology, and the precipitate particle dimensions were ~68 × 105 and ~53 × 94 nm^2^ (Figure 10a,c). Spherical precipitates were of diameters ~49 and ~52 nm (Figure 10e,g)). These precipitates were undissolved (Nb, Ti) (C, N) particles which could be confirmed by EDS analysis (Figure 10b,d,f,h).

**Figure 11:**
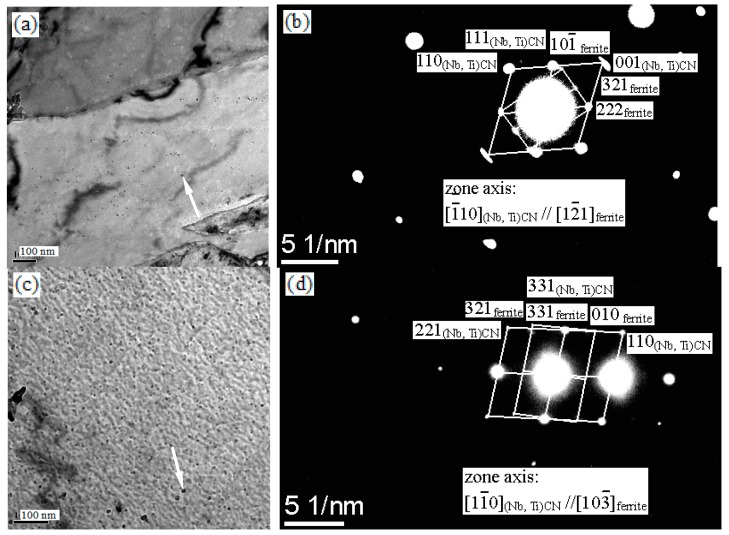
The presence of a dark precipitate (denoted by an arrow) particle and its schematic illustration of SAED held at (**a**,**b**) 600 °C; (**c**,**d**) 750 °C.

Figure 11a,b shows the selected area electron diffraction (SAED) from the particles dispersed in the ferrite matrix when the sample was isothermally held at 600 °C. The precipitate particle was fcc (Nb, Ti) (C, N) and was continued to be fcc (Nb, Ti) (C, N) at the 750 °C holding temperature, as confirmed by SAED (Figure 11c,d)). In fact, it was possible for the presence of complex carbides and co-precipitation of carbides and copper particles, which were reported in the literature [23]. In addition to carbonitride (Ti, Nb) (C, N), the remaining microalloying elements (Cu, V, etc.) still dissolved or precipitated in the matrix, which were confirmed experimentally by EDS analysis (Figure 10b,d,f,h).

## 4. Discussion

### 4.1. Microstructural Evolution

The specimens were isothermally held after two-stage deformation, which resulted in finer prior austenite grain size. PF, GB, and BF formed successively during cooling-isothermal treatment [23]. A uniform fine-grained structure contributed to the improvement in hardness. Isothermal holding temperatures were close to the critical temperature Ae1. Ferrite-grain coarsening occurred with increasing isothermal holding temperature at temperatures below Ae3 (Figure 5a–d). Finer ferrite grain size was a result of lower isothermal holding temperature, which led to more nucleation. Therefore, smaller ferrite grain size was obtained when specimens was isothermally held at 550 °C (Figure 5a and Figure 6a, Table 1). Solid-state transformations theory could be used to explain the formation of PF. According to the ferrite growth kinetics, the transformation of the ferrite is controlled by diffusion. The growth of the ferrite grain size requires long-range diffusion and the growth rate is related to temperature [24]. At the same time, a large amount of PF be appeared when the specimen was isothermally held at 800 °C (Figure 5f and Figure 6f, Table 1) [23]. Therefore, the PF was coarsest at 800 °C [2]. The decrease of the amount of the ferrite and fine ferrite grains was one reason for the increase of hardness. The hardness reached a maximum at a holding temperature of 600 °C and was related to a finer grain structure because 600 °C was close to Ar1 due to cooling. As mentioned above, a rather coarse-grained structure was obtained and the amount of PF increased to a certain degree when the specimen was isothermally held at 700 °C which led to decreased hardness. This was because that 700 °C is close to Ar3 at which austenite begins to transform to ferrite during the cooling process. The effect of boundary strengthening became weaker. This was the main reason for lowest hardness value. Increasing isothermal holding temperature increased the ferrite grain size from ~7.03 μm to 10.26 μm, and the amount of ferrite increased (Table 1). It was because that 800 °C was outclasses by Ar3 (Figure 2). As a result, hardness value decreased again.

Bainite nucleated on parent austenite grain boundaries, and its growth was completely contained within the parent austenite grain. At lower temperature, water cooling after isothermal holding resulted in the formation of BF free of internal carbides due to the low carbon content of the experimental steel. At higher temperatures, GB transformation occurred in despite of the fact that the time was not enough to nucleate due to water cooling. GB contained equiaxed, island-shaped MA constituents. MA islands (the mixtures of brittle martensite and residual austenite) affected hardness. Hardness increased to a maximum value at a holding temperature of 600 °C and then decreased rapidly to 650 °C and decreased gently at temperatures of 650–800 °C.

A prior ferrite core formed because 800 °C was under Ae3 when the specimen was isothermally held at this temperature. Ferrite/austenite boundary would move to austenite during the subsequent cooling. Carbon-rich (alloy-rich) islands would be enwrapped by ferrite in the final microstructure, and a significant amount of MA islands at a holding temperature of 800 °C were obtained. GB also has a slight strengthening effect in the experimental steel.

### 4.2. The Precipitation Behavior

Hardness is closely related to precipitation strengthening and grain refinement. When carbide forming elements such as Ti, Nb, V, Cr, and Mo were added to carbon steels, it has been frequently reported that alloy carbides were nucleated repeatedly at a growing ferrite/austenite boundary during ferrite transformation, leading to the formation of precipitates in rows parallel to ferrite/austenite boundary When carbide-forming elements, such as Ti, Nb, V, Cr, and Mo, were added to carbon steel, alloy carbides nucleated at the growing ferrite/austenite boundary during the ferrite/austenite transformation process. As the grain boundaries progressed, precipitated phases were formed parallel to the ferrite/austenite boundary [25]. When the sample was isothermally held at 550 °C, undissolved carbides could be found, but the fine dispersed precipitate phase was still difficult to observe (Figure 9a). Therefore, an extrapolation was possible that the precipitation began after 600 s at 600 °C. This could be verified by the presence of fine dispersed precipitates at a holding temperature of 600 °C (Figure 9b). The change in hardness is determined by the contribution of precipitation strengthening and matrix (mainly BF).

Deformation can affect the precipitation reaction kinetics. Precipitation can occur under the thermal simulation conditions. A few large-sized precipitates were undissolved (Nb, Ti) (C, N) particles (Figure 10a–h), even though the specimens were deformed and subsequently cooled and isothermally held. Carbonitrides were the main precipitate that affects their strength, even at low nitrogen levels (45 ppm).

The present steel contained alloying elements, such as titanium and vanadium. Yen et al. [26] reported that in the initial stages of the isothermal transformation, fine plate-like TiC particles form and these appear as Baker–Nutting (BN) OR and are related to the ferrite matrix. Miyamoto et al. [25] reported that VC maintains Baker-Nutting (B-N) orientation with ferrite and precipitates parallel to the austenite/ferrite phase interface. (Nb, Ti) (C, N) particles are shown in Figure 11. In general, the MX precipitates usually adapted the BN orientation relationship with the ferrite matrix due to the smaller forming energy barrier.

The precipitation strengthening effect was weakened by coarse precipitation and undissolved Nb/Ti carbonitrides according to the Ashby–Orowan model [27]. These precipitates should be insignificant not only after isothermal holding at 550 °C, 600 °C, 700 °C, and 800 °C, but also during the entire thermal simulation experiment. The Ashby–Orowan model could be used to accurately calculate the strengthening contribution of 3–30 nm size precipitated phases [27]. Table 3 presents the volume fraction, the average size, and the strengthening contribution of precipitates. The combination of small particle radius and small interparticle spacing has a significant strengthening effect. Combined with the refinement of ferrite grains, it has a comprehensive strengthening effect. The hardness reached a maximum at a holding temperature of 600 °C. (Figure 4). Finely-dispersed precipitates (Nb/Ti carbonitrides) played a strengthening role. These precipitates became coarse at a holding temperature of 800 °C. The average size of the precipitates increased from 3.14 nm to 4.83 nm when the sample isothermal temperature was changed from 600 °C to 800 °C. Interparticle spacing of precipitates also increased with increasing isothermally held temperatures. For example, it increased from 73 to 97 nm when the specimens were isothermally held at 700 °C and 750 °C (Figure 9d,e). These resulted in a decrease in precipitation strengthening.

The kinetic analysis of precipitation during TMCP were completed in a low-carbon microalloyed steel, and there was a bay (local maximum) in the precipitation-time–temperature (PTT) diagram [28,29,30]. It was considered in the present study that precipitation occurred after 600 s at temperatures between 600 °C and 800 °C. Isothermal holding temperature influences the extent of precipitation. As mentioned previously, after isothermal holding at 700 °C for 30 s, interphase precipitation began to occur [23]. The kinetics of precipitation at 750 °C were enough to nucleate many precipitates (Figure 9e, Figure 11c). Therefore, a high hardness peak was achieved by effective precipitation strengthening (Figure 4). It was speculated that the precipitation behavior became faster at the temperature of 800 °C. The precipitation might be complete or close to complete. During higher isothermal temperature processes, the size and spacing of the precipitated phases increased (Figure 9f). The coarsening of the precipitated phase significantly weakened the strengthening effect, but the precipitated phase still hindered the growth of the grains to a certain extent. As a result, the Vickers hardness of the specimen exhibited a peak-to-valley variation.

The resultant hardness of the experimental steel after precipitation was a direct result of type, size, shape, and distribution of precipitates. The precipitates are mx-type precipitates fcc (Ti, Nb) (N, C) particles. Mo-rich precipitates were not found by EDS analysis and SAED because of low molybdenum content (Figure 10 and Figure 11). The addition of Mo increased the hardenability of the steel, thereby increasing the hardness. On the other hand, the addition of Mo reduced the row spacing and the size of the interphase precipitates [31]. Therefore, it could be considered that the precipitation of Mo on the austenite/ferrite interface and the subsequent coarsening process after the passage of the transformation interface have a beneficial effect on reducing the IP spacing [31]. Mo reduced the interface energy to increase the nucleation rate, thereby reducing the size of carbide precipitates and delaying its coarsening [31]. As a consequence, rather high hardness values were attained.

## 5. Conclusions

This paper analyzed the formation of BF, GB, PF, and MA islands and the distribution of precipitates after different isothermal treatments through the isothermal process experiments of X90 pipeline steel, and established the effect mechanism of isothermal process on the evolution and precipitation behavior of the microstructure of experimental materials.

(1) The hardness reached maximum value (HV0.5 401) at 600 °C holding temperature and was related to a finer grain structure. This was also related to the strengthening effect of GB. The strengthening effect of precipitates was remarkable due to the combination of small particle radius and small interparticle spacing, and this was the prime reason of hardness variation. An extrapolation was possible showing that the precipitation began after 600 s at 600 °C. The precipitates were fcc (Ti, Nb) (N, C) particles, and they belonged to the MX-type precipitates.

(2) When the sample was isothermally held at 700 °C, coarsened ferrite grains were obtained, and the amount of PF increased, which led to decreased hardness (HV0.5 312). Precipitation kinetics showed that when isothermally held at 750 °C, it was sufficient to nucleate many precipitated particles. Therefore, high hardness was obtained by effective precipitation strengthening.

(3) Average size of precipitates increased from 3.14 to 4.83 nm when the specimens were isothermally held at 600–800 °C. Interparticle spacing of precipitates also increased with increasing isothermally held temperatures. These resulted in decrease in precipitation strengthening. At the same time the amount of PF increased considerably and ferrite grain size grew larger. Thus, hardness decreased continuously. The hardness reached its minimum value (HV0.5 295) at a holding temperature of 800 °C.

## Figures and Tables

**Table 1:** The volume fractions of microstructure constituents and PF grain size.

Specimens (°C)	GB (%)	BF (%)	PF (%)	MA Islands (%)	Average Grain Size of PF (μm)
550	36.76 ± 3.9	63.86 ± 3.1		1.39 ± 1.6	7.03 ± 0.6
600	37.75 ± 4.2	60.84 ± 4.5		1.65 ± 0.4	7.19 ± 0.15
650	20.37 ± 3.7	77.64 ± 2.8		1.03 ± 0.5	7.24 ± 0.54
700	28.37 ± 3.2	70.57 ± 2.9		1.27 ± 0.6	7.52 ± 0.40
750	-	98.64 ± 4.5		1.27 ± 1.2	8.90 ± 0.94
800	-		92.46 ± 3.7	7.54 ± 1.0	10.26 ± 0.13

**Table 2:** The hardness of the microstructural constituents.

Specimens (°C)	GB (Hv 0.5)	BF (Hv 0.5)	PF (Hv 0.5)	MA Islands (GPa)
550	216.2 ± 12.8	227.1 ± 12.1	175.9 ± 17.0	4.027 ± 0.68
600	337.2 ± 27.0	346.7 ± 20.1	266.3 ± 7.3	7.817 ± 0.75
650	221.0 ± 14.8	299.6 ± 38.8	189.6 ± 9.7	6.334 ± 0.40
700	229.1 ± 16.6	207.9 ± 6.6	209.6 ± 6.6	4.349 ± 0.57
750	178.0 ± 6.0	183.7 ± 14.3	155.6 ± 14.0	3.950 ± 0.42
800	300.5 ± 28.5	195.3 ± 25.2	138.5 ± 5.8	3.756 ± 0.72

**Table 3:** The strengthening contribution of precipitates.

Specimens (°C)	Volume Fraction (%)	Average Size (nm)	Strength Increase (MPa)
550	-	-	-
600	0.698 × 10^−3^	3.14	101.38
650	0. 613 × 10^−3^	4.47	78.08
700	0. 635 × 10^−3^	4.74	75.54
750	0. 623 × 10^−3^	4.74	75.80
800	0. 332 × 10^−3^	4.83	54.70
